# Fighting against the common enemy of COVID-19: a practice of building a community with a shared future for mankind

**DOI:** 10.1186/s40249-020-00650-1

**Published:** 2020-04-07

**Authors:** Xu Qian, Ran Ren, Youfa Wang, Yan Guo, Jing Fang, Zhong-Dao Wu, Pei-Long Liu, Tie-Ru Han, Members of Steering Committee, Society of Global Health, Chinese Preventive Medicine Association, Zong-Fu Mao, Yu Jiang, Tian-Ping Wang, Jia-Hui Zhang, Qing-Min Zhang, Zhao-Yang Zhang, Hong-Ning Zhou, Feng Chen

**Affiliations:** 1 School of Public Health/Global Health Institute, Fudan University, Shanghai, 200032 China; 2 Global Health Center, Dalian Medical University, Dalian, 116021 China; 3 Global Health Institute, School of Public Health, Xi’an Jiaotong University, Xi’an, 710061 China; 4 Global Health Center, School of Public Health, Beijing University, Beijing, 100871 China; 5 Kunming Medical University, Yunnan, 650500 China; 6 Zhongshan School of Medicine, Sun Yat-Sen University, Guangzhou, 510080 China; 7 Society of Global Health, Chinese Preventive Medicine Association, Beijing, 100013 China

**Keywords:** COVID-19, SARS-CoV-2, Outbreak, Pandemic, Fighting, Preparedness, International health regulation, Quarantine

## Abstract

The outbreak of coronavirus disease 2019 (COVID-19) has caused more than 80 813 confirmed cases in all provinces of China, and 21 110 cases reported in 93 countries of six continents as of 7 March 2020 since middle December 2019. Due to biological nature of the novel coronavirus, named severe acute respiratory syndrome coronavirus 2 (SARS-CoV-2) with faster spreading and unknown transmission pattern, it makes us in a difficulty position to contain the disease transmission globally. To date, we have found it is one of the greatest challenges to human beings in fighting against COVID-19 in the history, because SARS-CoV-2 is different from SARS-CoV and MERS-CoV in terms of biological features and transmissibility, and also found the containment strategies including the non-pharmaceutical public health measures implemented in China are effective and successful. In order to prevent a potential pandemic-level outbreak of COVID-19, we, as a community of shared future for mankind, recommend for all international leaders to support preparedness in low and middle income countries especially, take strong global interventions by using old approaches or new tools, mobilize global resources to equip hospital facilities and supplies to protect noisome infections and to provide personal protective tools such as facemask to general population, and quickly initiate research projects on drug and vaccine development. We also recommend for the international community to develop better coordination, cooperation, and strong solidarity in the joint efforts of fighting against COVID-19 spreading recommended by the joint mission report of the WHO-China experts, against violating the International Health Regulation (WHO, 2005), and against stigmatization, in order to eventually win the battle against our common enemy — COVID-19.

## Background

A sudden outbreak of coronavirus disease 2019 (COVID-19) caused by infection with severe acute respiratory syndrome coronavirus 2 (SARS-CoV-2) virus has happened since December 2019 in Wuhan City, Hubei Province, a central city in the People’s Republic of China, where transportation is enormously convenient to connecting all other places in China and overseas [1, 2]. As of 7 March, 2020, a total of 80 813 confirmed cases reported in all provinces of China, and 21 110 cases reported in 93 countries/territories/areas of six continents [3]. In particular, some cases have been confirmed in African countries, such as Algeria, Egypt, and Nigeria [3]. This is the biggest infectious disease outbreak in China ever since 1949, the year of founding the People’s Republic of China. It is the biggest battle since the disease is spreading so fast with high prevalence, and the prevention of the transmission has involved all people in the country [4]. While at global level, the strategy and coordinating mechanism to control COVID-19 need to be set down as soon as possible [5], in particular, three questions need to be addressed as (i) how to take the emergency response actions effectively in different countries? (ii) how to mobilize resources quickly with strategic ways? and (iii) how to encourage people proactively and orderly to participate in this battle against COVID-19 from different regions of the world?

## Lessons from the battle against COVID-19 in China

In order to address three aforementioned questions, the lessons from China in the battle against COVID-19 need to be understood clearly in the following three aspects:

### Traditional epidemiological approaches effectively control the transmission

Professionally speaking, three steps are necessary to taken once an infectious disease outbreaks in certain regions, including controlling infectious sources, blocking the transmission routes, and protecting the susceptive population [6]. While, as COVID-19 spreading so fast and people’s travelling so frequent during the Chinese New Year (Spring Festival) season, it cannot control effectively if only taking the normal or general countermeasures [7]. Therefore, the Chinese government has quickly taken actions to contain its transmission inside China, including detecting the disease early, diagnosis and reporting early, isolating and treatment of cases early, tracing all possible contacts, persuading people to stay at home, and promoting social distancing measures commensurate with the risk, etc., based on the current knowledge about epidemiological features and transmission patterns of COVID-19.

### Response strategies coping with local conditions

In dealing with the outbreak, China has been adopting the way of tailoring interventions into local settings, from quickly finding each infected person, tracing close contacts and placing them under quarantine, to promoting basic hygiene measures to the public, such as frequent hand washing, cancelling public gathering, closing schools, extending the Spring Festival holiday, delaying return to work, and to the most severe measure of city lockdown of Wuhan [8, 9]. By adapting response strategies to the local context, it may avoid blockading the city when it is not needed, and also prevent from a major outbreak without taking any action.

### Mobilizing resources quickly to support the emergency responses

Under the strong leadership of the Central Government of China, the mobilization for the emergency responses has been effectively promoted in following ways. Firstly, a Joint Prevention and Control Mechanism of the State Council has established involving 32 Ministries, with subgroups on control of outbreak, medical rescue, scientific research, information and communication, international cooperation, logistics, and frontline coordination [10]. This multi-sectoral cooperation mechanism at high level is to ensure the facilities and supplies have been well arranged to support the emergency responses in all provinces, with focus on the Hubei Province, for example, more than 10 mobile hospitals and two big hospitals with each one having the capacity of holding more than 1000 beds have been built within 10 days. Secondly, more than 40 000 medical professionals from other provinces or military institutions have been dispatched to Hubei Province to implement emergency responses, including medical care and treatment, epidemiological investigations, environmental sterilization for disinfection, and data and information management to support the policy making.

### Encouraging people proactively and orderly participate in this battle against COVID-19

It is important to protect the community from exposure to the infection, all residents in the potential risk areas were encouraged to stay at home, which is an effective way to block the transmission routes. Local community health workers and volunteers, after the specific training, proactively participate in screening the suspicious infections, and help in implementing proper quarantine measures by providing support services, such as driving patients to the mobile hospitals [8]. All those activities logistically managed at the community level.

At the same time, from medical care side, the medical doctors and nurses worked very hard in the hospitals, to screen the suspected cases, provide medical care for the confirmed cases, and taking emergency response to rescue severe patients to reduce the fatality. While epidemiologists working in centers for disease control and preventions provided the statistical results for the dissemination of epidemiological data correctly, and provide the well-prepared datasets for the decision makers for coordination of necessary resources, and many health workers investigate the suspected contactors for quick medical quarantine of the suspected cases at the community level.

### Preventing the pandemic of COVID-19

With the conceptualization on building a community with a shared future for mankind proposed by Chinese President Xi Jinping in 2013 [11], Chinese people have taken following actions to prevent the pandemic of the diseases: (i) sharing the sequences of SARS-Cov-2 virus with the World Health Organization (WHO) and other countries which are important information for other countries to prepare the tests for screening and diagnosis, (ii) all epidemiological data with clinical treatment in China has been published in the international journals, (iii) prevent spreading of the disease by traveling ban in Wuhan, (iv) medical quarantine has been performed for all suspected contactors, (v) body temperature measuring facilities were equipped in all railway stations and airports, etc. In order to take very strict contain measures for COVID-19 outbreak tailored to local settings, the travelling ban was executed in Wuhan, and encouraging no gathering and less travelling in other cities out of Hubei Province. Those actions were implemented by strong coordinating of the Chinese government in cooperation with local residents. To date, the epidemiological data has showed more than thousands of people have been protected from the infections, and increasing pattern of the transmission has been suppressed significantly in China [12].

## Challenges in fighting against COVID-19

The fighting against COVID-19 has been lasting almost two months, and the time left for people outside of China to prepare the countermeasures has been narrowed quickly. To date, we have found it is one of the greatest challenges to human beings in fighting against COVID-19 in the history, since the pathogen of SARS-CoV-2 is a new coronavirus, differed from either SARS-CoV or MERS-CoV in terms of biological characteristics and transmissibility [13].

Technically, we have little knowledge on the pathogen and pathogenesis, without specific effectively drugs or vaccine against the virus infection, which cause difficulties in rescuing the severe cases which account for about 20% of the infections. The transmission routes are not clear enough, although we currently understand that the respiratory transmission from human to human is the major transmission route, but other ways for transmission, such as gastrointenstinal transmission or aerosol propagation, is not so clear.

Administratively, implementing the locked down measures in such a big city with over 15 millions of people is not an easy task, with a lot of preparing works from different dimensions of municipal logistic management, to support the emergency response actions. Thus, the multi-administrative systems need to be coordinated collectively, guiding from the central government, with more resources gathering from various places all over the country.

Globally, the information sharing is so important, including patients’ information sharing to trace the suspected cases to protect more people as quickly as possible, genome sequences information sharing to prepare the diagnostics as quickly as possible, and treatment schemes sharing to rescue more severe cases. The WHO declared the Public Health Emergency of International Concern based on the International Health Regulation (2005) in the early time of the outbreak of COVID-19, as it is an extraordinary event to constitute a public health risk to the states through the international spread of disease, and to potentially require a coordinate international response [14]. All actions to strengthen surveillance and response systems on infectious diseases need to put emphasis on resources limited countries, such as Southeast Asia and African countries [15].

## Recommendations

With understanding more about the nature of COVID-19, it is necessary to understand clearly the current challenges against COVID-19 become increasing, not only to China but also to the world. In order to take quick actions to early prepare the battle against COVID-19 and better allocate enough health resources from the world, the recommendations are as follows:

### Coordinating interventions and resources mobilization globally

#### Preparedness in low and middle income countries

WHO has identified 13 African countries at the top-risk affected by COVID-19 but with limited resources against COVID-19, including Algeria, Angola, Cote d’Ivoire, the Democratic Republic of the Congo, Ethiopia, Ghana, Kenya, Mauritius, Nigeria, South Africa, Tanzania, Uganda and Zambia. These countries have direct links or greater numbers of people travelling to/from China [15]. The preparing works on response to the imported cases need initiated as soon as possible with the assistance of WHO as well as developed world. The major preparing works are to prepare enough facilities for use in hospitals, such as test kits, facemasks, and personal protective equipment (PPE), to prepare the quarantine measures in each gate of the traveling venues, and to prepare information communication, etc. The emergency response mechanism on multi-sectoral cooperation needs to be established once the first case has been detected.

#### Intervention and coordination globally

The fast spreading of COVID-19 to more than 90 countries/territories, with some cluster cases occurred in a few countries, demonstrated that this new disease has higher transmissibility compared with SARS and MERS. The nature of high transmissibility for COVID-19 requires us to (i) prepare the battle globally as soon as possible, by taking the advantage of the time window opened by Chinese battle against COVID-19, (ii) invest more weapons or tools against the diseases by better global coordination, and (iii) take proper quarantine measures globally [16]. We are able to win the battle only if our actions are coordinated better at a global level.

#### Resources mobilization globally

One of lessons learnt from the battle in Wuhan is the speed of resources gathering against COVID-19 outbreak could not catch up the speed of the coronavirus spreading in early stage of the outbreak, and it is in need of support or assistances from outside of epicenter, including medical doctors, nurses, and facilities of PPE used in hospitals, and facemasks for residents. The strong support from outside of epicenter quickly to ensure all infectious sources either controlled through quarantine measures or treated in the specialized hospitals. Therefore, for those countries with weak health system, it is so urgent to get help from other parts of the world. WHO needs to mobilize its certified global emergency medical teams to get ready to be dispatched to other countries where health workers are in short supply while an outbreak occurs.

### Jointly fighting against common enemy ─ COVID-19

As said by WHO Director-General in the news press on Public Health Emergency of International Concern declaration that “this declaration is not a vote of no confidence in China, our greatest concern is the potential for the virus to spread to countries with weaker health systems.” Therefore, international community needs to work together to prepare for the containment of COVID-19 transmission and spreading in other countries, under the scenario that more countries may be affected by the new coronavirus [17]. These containment works have to quickly take readiness on active surveillance, early detection, isolation and case management, contact tracing and prevention of onward spread of COVID-19.

Therefore, at this stage, with more countries having confirmed more and more COVID-19 cases, all countries need work together on the following global actions on:

(i) fighting against COVID-19 spreading, including sharing the information of the disease transmission and epidemiological knowledge, sharing the experiences on case management and treatment approaches both for severe cases or light symptoms, and sharing new technologies or strategies to contain the transmission;
(ii) fighting against violating International Health Regulation, by following the WHO’s authoritative advices which called on all countries to implement decisions that are evidence-based and convincing. We need to improve our quarantine measures to replace the disconnection of international traveling and trade restrictions, with an assistance of the improved active surveillance systems and AI-based technology to trace the contactors;
(iii) fighting against stigmatization, since the stigmatization is always present when the disease outbreak and people facing the sudden attack of this kind of epidemic. These phenomena on stigmatization may be at a scale of epicenter areas, or may be at a country and regional scale, and even at global scale. Thus, we need fight with the real and common enemy which is the new coronavirus, rather than the infected people. The international community needs the solidarity and sympathy to start the battle against the common enemy – the new coronavirus, as well as against stigmatization at the same time.

### Global cooperation in priority settings

By considering COVID-19 is spreading so fast which causes difficulties in containing the disease, we, as a community of shared future for mankind, need better coordination in global cooperation and further improvement in the multi-sectoral cooperation in order to quickly take response and prevent from the pandemic [18]. In addition, we also need better coherence of our resources with more international partners, at least, we can quickly improve our priority settings in sharing information and data, on research priority settings, on surveillance and response to outbreaks at a global level.

#### Cooperation on sharing information and data

In order to quickly share the information and datasets for countermeasures, the actions on fast and open reporting of outbreak data and sharing of virus samples, genetic information, and research results are encouraged for all international communities, non-governmental organizations (NGOs), as well as governmental institutions around the world. Through regional and country office of WHO, more preventive information against COVID-19 can be disseminated to the public in the vulnerable countries.

#### Coordination on surveillance and response

With understanding the importance of human health in the planet, multi-sectoral and multi-lateral cooperation against COVID-19 pandemic are recommended at global level. Particularly, the scientific communities, governments and NGOs in different fields, such as public health, agriculture, ecology, epidemiology, governance planning, science, and many others need to collaboratively prevent future outbreaks, with better coordination. The secretary of the United Nations need take the responsibility to coordinate the actions on protecting the planetary health by systematic approaches, such as EcoHealth, One Health, Planetary Health and Urban Health, and making sure public resources are worthwhile investing in strengthening surveillance and response systems for preventing future outbreaks of emerging infectious diseases.

#### Coherence on research priority settings

We urgently encourage all governments and international foundation to support short-term and emergency response-related research projects to improve our understanding of the causes, risks, infectiousness, and threats of a pandemic [19]. Health institutions at international level should be encouraged to organize the research priority settings on preventing the pandemic or averting the emergence of the disease. International conservation organizations start to take investigations on types of wildlife-pathogens interactions affecting human health. International environmental agencies can initiate researches on unsustainable transformations of natural environments and ecosystems that provide life-supporting services for our health.

## Conclusions

To summarize, COVID-19 is a new disease that has caused great impacts to the people’s daily life extraordinarily. We, as a community of shared future for mankind, need to take collectively and quickly strong emergency responses as a battle against our common enemy, the new coronavirus, not only in China but also in the world. All partners of international community and country leaders are encouraged to proactively take strategic actions as soon as possible to fight the COVID-19 together. Hard times will end finally, and we will meet each other in the blooming spring soon.

## Data Availability

All data supporting the findings of this study are included in the article.

## Abbreviations

COVID-19: Coronavirus disease 2019
SARS-CoV-2: Novel severe acute respiratory syndrome coronavirus
SARS-CoV: Severe acute respiratory syndrome coronavirus
MERS-CoV: Middle East respiratory syndrome coronavirus
NGOs: Non-governmental organizations
WHO: World Health Organization
PPE: Personal protective equipment
